# Transcriptional Regulators of Claudins in Epithelial Tight Junctions

**DOI:** 10.1155/2015/219843

**Published:** 2015-04-08

**Authors:** Niamat Khan, Abdul R. Asif

**Affiliations:** ^1^Institute for Clinical Chemistry/UMG-Laboratories, University Medical Center Göttingen, Robert-Koch-Straße 40, 37075 Göttingen, Germany; ^2^Department of Biotechnology & Genetic Engineering, Kohat University of Science and Technology, Kohat 26000, Khyber Pakhtunkhwa, Pakistan

## Abstract

Human gastrointestinal tract is covered by a monolayer of specialized epithelial cells that constitute a protective barrier surface to external toxic and infectious agents along with metabolic and digestive functions. Intercellular junctions, among epithelial cells, such as desmosomes, adherens, gap, and tight junctions (TJs), not only provide mechanical integrity but also limit movement of molecules across the monolayer. TJ is a complex structure composed of approximately 35 different proteins that interact with each other at the apical side of two adjacent epithelial cells. Claudin family proteins are important members of TJ with so far 24 known isoforms in different species. Claudins are structural proteins of TJ that help to control the paracellular movement by forming fence and barrier across the epithelial monolayer. Altered function of claudins is implicated in different form of cancers, inflammatory bowel diseases (IBDs), and leaky diarrhea. Based on their significant role in the molecular architecture of TJ, diversity, and disease association, further understanding about claudin family proteins and their genetic/epigenetic regulators is indispensable.

## 1. Introduction

Epithelial monolayer (EM) is the largest body tissue lining many organs in the human body. In the intestine, EM provides protection to the internal body from toxic and infectious agents while at the same time it facilitates absorption of digested food and water from the gut. Epithelial monolayer integrity and paracellular transport are the important features that can be protected and maintained with the help of epithelial barrier function [1]. Epithelial cells are connected with each other by four types of junctions, that is, desmosomes, gap junctions, adherens junctions, and TJs [2–4]. Tight junctions are impermeable and control the movement of molecules and ions via a paracellular pathway. Until recently, tight junction functions were categorized as “fence” as they separate the apical and basolateral cell surface domain defining cell polarity or a “barrier” due to their control over solutes and liquid flow through the paracellular space between the epithelial cells [5–8]. However TJs are not restricted to the fence and barrier function but have been defined to participate in signal transduction processes, gene expression, cell proliferation, and differentiation [9–11]. Various unidentified external and internal regulators impair the normal function of TJs causing loss of water and solute in the passive manner that leads to leaky-flux watery diarrhea. The unwanted invasion of noxious luminal antigens prolongs the existence of mucosal inflammatory processes [12].

Tight junction (TJ) is a complex structure constituting of growing numbers of components, including integral membrane proteins (claudins, occludin, and junctional adhesion molecules “JAMs”) and peripheral membrane proteins. The peripheral membrane proteins include (1) scaffold PDZ (postsynaptic density protein (PSD95), Drosophila discs large tumor suppressor (Dlg1), and Zonula occludens-1 protein (ZO-1)), multi-PDZ domain protein-1 (MUPP-1), and membrane-associated guanylate kinase (MAGI-1); (2) no-PDZ expressing proteins such as cingulin, symplekin, atypical protein kinase C, Ras-related protein Rab-3B (Rab-3b), Ras-related protein Rab-13 (Rab-13), phosphatase and tensin homolog (PTEN), and 7H6 antigen; (3) cell polarity molecules ASIP/PAR-3, partitioning defective 6 homolog alpha (PAR-6), and PALS1-associated TJ protein (PATJ) [13, 14]. Besides these proteins, tricellulin protein has recently been identified at the epithelial cell junctions with involvement in the barrier function [15].

*Claudin family* so far includes 24 reported members in different types of mammalian cells; among them 21 are known components of TJ in EM in the kidneys, liver, brain, and intestine [25]. These are involved in various physiological processes such as regulation of paracellular permeability and conductance. Claudins are found in homo and heterotypic manner in single TJ [13, 26]. They can be divided into two main categories, “pore-sealing” and pore-forming claudins. Claudin-1, -3, -4, -5, -7, and -19 are known as pore-sealing claudins and an increased expression of these claudin proteins leads to increased tightness of EM and increased transepithelial electrical resistance (TEER) and decreases solute permeability across the monolayer [27–31]. On the other hand, claudin-2 and -15 are considered as the “pore-forming claudins,” because of their ability to form paracellular anion/cation pores as well as water channels and therefore they decrease epithelial tightness and increase solute permeability [13, 32].

Epithelial barrier dysfunctions occur in inflammatory bowel diseases (IBDs) like Crohn's disease (CD) or ulcerative colitis (UC) that contribute to leaky-flux diarrhea, that is, loss of solutes and water in increased amount dependent upon the components of TJ proteins. Downregulation of pore-sealing claudins (e.g., 4, 5, and 8) while upregulation of pore-forming claudin-2 is observed in active Crohn's disease patients [33, 34]. Similarly downregulation of pore-sealing claudin-4 is also associated with UC disease [34]. Numerous studies have reported leaky diarrhea in patients undergoing immunosuppressive therapy after organ transplantation [35–38]. Recently our group has reported mycophenolic acid- (MPA-) mediated increased expression of myosin light chain kinase (MLCK), myosin light chain-2 (MLC-2), and MLC-2 phosphorylation and redistribution of ZO-1 and occludin in Caco-2 and in HEK-293 cells [39, 40] as a possible mechanism of diarrhea in patients undergoing immunosuppressive therapy. Transcription factors (TFs) play an important role in the gene regulation at the promoter level working either as an activator or as a repressor of a specific gene. The current review will focus major claudins family members (Table 1) and their regulators, which alter claudins gene activity at promoter level and therefore modulate TJs structure and function.

## 2. Claudin-1

Claudin-1 protein is a key constituent of TJs and its altered expression is reported in a variety of cancers, most prominently colorectal cancers [13, 17, 41, 42]. Promoter region (−1160 bps to −850 bps) of claudin-1 consists of putative binding sites for caudal-related homeobox (cdx-1, -2), GATA4, and T-cell factor/lymphoid enhancing factor-1 (Tcf/Lef-1) transcription factors. There is a direct correlation between claudin-1 and cdx-2 expression in human colon cancer patient [17]. Cdx-2 is a homeobox domain-containing nuclear transcription factor that plays an important role in intestinal development by regulating the proliferation and differentiation of intestinal cells [43–45], and it is expressed in all cells along the crypt villus axis. Cdx-2 transcriptional activity is controlled through mitogen-activated protein kinase/extra cellular signal regulated kinase pathway (MAPK/ERK pathway) which phosphorylates it at ser-60 position and resultantly reduces cdx-2 transcription activity in crypt and lowers villus cells. On the other side, cyclin-dependent kinase 2 (CDK2) phosphorylates cdx-2 at Ser-281 which coordinates cdx-2 polyubiquitination and degradation by the proteasome [43, 46–49].

Specificity protein-1 (Sp-1) is the first identified transcription factor of specificity protein/Krüppel-like factor (Sp/XKLF) family, consisting of 785 amino acids (aa) with molecular weight of 100 to 110 kDa. Sp-1's DNA binding domain is the most conserved among other domains of SP family members which consisted of Cy2His2 Zinc (Zn) fingers. Mutational analysis has revealed that Zn fingers 2 and 3 are essential for DNA binding activity [50]. Sp-1 binds to the GC-rich elements [51] that are common regulatory elements in the promoters of numerous genes. Sp-1 binds its individual binding sites as a multimer and is capable of synergic activation of promoters containing multiple binding sites [52] and regulates transcription by dynamically recruiting and forming complexes with many factors associated with transcription [53]. Normally Sp-1 has been described as a transcriptional activator but it can also act as a repressor [54]. Claudin-1 promoter region (−138 to −76 bp) contains Sp-1 binding site and a mutation in this region results in a significant loss of claudin-1 transcription [16].

## 3. Claudin-2

Claudin-2, also known as leaky protein, forms paracellular water channels in TJs and mediates paracellular transport of water molecules across the EM. EM permeability is enhanced by increased expression of claudin-2 in TJs. It is also involved in many signaling pathways, including vitamin D receptor, epidermal growth factor receptor (EGFR), and c-Jun N-terminal kinases (JNK) signaling pathways, and contributes to inflammatory bowel disease and colon cancer [33, 55–58].* Salmonella *infection facilitates bacterial invasion across the EM by inducing claudin-2 expression and altering its localization in TJs which is reversible by specific inhibitors (EGFR (Gefitinib) and JNK (SP600125)), making claudin-2 as a potential therapeutic target to prevent bacterial invasion and inflammation [59].

Interleukin-6 (IL-6) increases TJ permeability of Caco-2 monolayer from the basal side by inducing caludin-2 expression. IL-6 activates the mitogen-activated protein kinases/extracellular signal-regulated kinases (MEK/ERK) pathway by inducing phosphorylation of ERK and phosphatidylinositol 3′-kinase (PI3K/Akt) by phosphorylating Akt, which in turn enhances cdx-2 expression. In the claudin-2 promoter region (−1067 to −1), four cdx-2 (cdx-A, -B, -C, and -D), STAT, and nuclear factor-kappa-light-chain-enhancer of activated B cells (NF-*κ*B) putative binding sites are identified. IL-6 induced expression of claudin-2 can be reversed by using either specific inhibitors of MEK/ERK and PI3K/AKT pathways (U0126 (a MEK inhibitor) and LY294002 (a PI3K inhibitor)) or site directed mutagenesis in the putative cdx-2 binding sites in the promoter region of claudin-2 gene [18].

## 4. Claudin-3, Claudin-4, and Claudin-5

Both claudin-3 and claudin-4 are overexpressed in ovarian cancer. A Sp-1 binding site (−112 and −74 bps) in the promoter region of claudin-3 is crucial for its activation. Claudin-3 expression is significantly decreased at mRNA and protein levels, by knocking down the Sp-1 with siRNA, indicating an essential role of Sp-1 in claudin-3 activation [19]. Claudin-4 is mainly expressed in the EM of colon, renal tubules, mammary gland, and thyroid gland and is considerably raised in their cancers [60]. There are two known Sp-1 binding sites (between −105 and −49 bps) in the promoter region of claudin-4 [20].

Caludin-5 is mostly expressed in the TJs of EM of pancreatic acinar cells, alveolar lung cells, colon, and endothelial cells forming the blood-brain barrier and endoneurial blood-nerve barrier. In colonic regions, its expression is mainly involved in the paracellular sealing of TJs [33, 61–63]. Both downregulation and redistribution of claudin-5 can alter TJs structure leading to barrier dysfunction in active Crohn's disease [33]. Forkhead box (foxO) gene family members are potent transcriptional activators with four known members; foxO1 (also known as foxO1a), foxO3 (also known as foxO3a), foxO4, and foxO6 which bind to conserved consensus core recognition motif TTGTTTAC [64–66]. Four pairs of putative binding sites for foxO and tcf-*β*-catenin (Tcf-*β*-catenin act as a stabilizer) are identified in the three regions of claudin-5 promoter (region 1, position −2,906/−2,871; region 2, position −2,317/−2,287; region 3, position −1,103/−1,008). Both foxO1 and tcf-*β*-catenin interact with region 1 of the caludin-5 promoter to repress its transcription [21].

## 5. Claudin-7

Claudin-7 is expressed prominently in the biphasic type of synovial sarcoma of adults. E74-like factor 3** (**ELF3) belongs to E26 transformation-specific sequence (ETS) family of transcription factors and binds to the Ets binding site in the promoter region (−150 bps) of claudin-7 [22]. Members of ETS family are mainly involved in cell differentiation, proliferation, and cell transformation [67]. Regulation of the target genes by ETS factors depends upon their activation by MAPK and their association with other cofactors [68, 69]. An essential role of ELF3 is reported in epithelial cell differentiation [70–72] and small interference RNA (siRNA) treatment downregulates the claudin-7 expression validating the central role of ELF3 in claudin-7 activation.

## 6. Claudin-15

Claudin-15 is a pore-forming protein expressed in the EM of intestine, liver, and kidney tissues. Downregulation of claudin-15 decreases permeability of EM layer and can initiate IBD. Four putative binding sites (BS1-4) of transcription factor hepatocyte nuclear factor 4 alpha (hnf4*α*) are present in the (−693 to −47 bps) region of claudin-15 promoter [23]. Hnf4*α* is considered as an important regulator of EM barrier integrity and is involved in the regulation of metabolism, cell junction, differentiation, and proliferation of liver and intestine epithelial cells [73]. Both animal model and IBD patients' biopsy studies have shown that an altered expression of hnf4*α* directly influences the expression and distribution of claudin-15 [23].

## 7. Claudin-19

The kidney is responsible for the filtration of excretory material from the blood. However, 25–40% of filtered Na^+^ [74], 50–60% of filtered Mg^2+^ [75], and 30–35% of filtered Ca^2+^ [76] are reabsorbed into the body by thick ascending limb, the loop of Henle. Claudin-16 and -19 play a main role in the regulation of Mg^2+^ reabsorption and loss of either claudin-16 or -19 leads to excessive renal waste of Mg^2+^ [77]. Four putative transcription factor (not characterized, AP2, NF-E, and Sp-1) binding sites are located between −139 and −75 in the promoter region of mouse claudin-19. However, only Sp-1 is described for having an important role in the expression of claudin-19 and a mutation in Sp-1 binding site significantly reduces the claudin-19 expression [24].

## 8. Conclusion

Tight junctions play an important role in the regulation of paracellular movement of molecules across the EM, impart mechanical strength, maintain the polarity of cells, and prevent the passage of unwanted molecules and pathogens through the space between the plasma membranes of adjacent cells. The efficiency of the junction in preventing ion passage increases exponentially with the number of strands of claudins family proteins which are having important role in the structure as well as controlling paracellular movement across the tight junctions. Altered expression of claudins family proteins in TJs plays a key role in numerous abnormalities like cancers, IBDs, and leaky diarrhea and a better understanding of their regulatory mechanism could help in designing innovative therapeutic strategies.

## Figures and Tables

**Table 1:** Regulators of claudins.

TJ proteins	Regulator	Promoter binding region	Expression/reference
Claudin-1	Sp-1	−138 to −76 bp	↑ [16]
	cdx-2	−1160 to −850 bp	↑ [17]
Claudin-2	cdx-2	−1067 to −1 bp	↑ [18]
Claudin-3	Sp-1	−112 to −74 bp	↑ [19]
Claudin-4	Sp-1	−105 to −49 bps	↑ [20]
Claudin-5	FoxO1	−2,906 to −2,871 bps	↓ [21]
Claudin-7	ELF-3	−150 bps	↑ [22]
Claudin-15	Hnf4*α*	−693 to −47 bps	↑ [23]
Claudin-19	Sp-1	−139 to −75 bps	↑ [24]

Note: arrow (↑) = upregulation, arrow (↓) = downregulation.
